# Integrated bioinformatics and *in silico* approaches reveal the biological targets and molecular mechanisms of 1,25-dihydroxyvitamin D against COVID-19 and diabetes mellitus

**DOI:** 10.3389/fnut.2022.1060095

**Published:** 2022-12-02

**Authors:** Fanqiang Zeng, Yongli Xu, Chaoling Tang, Zhigang Yan, Chaohe Wei

**Affiliations:** ^1^Department of Pharmacy, Guigang City People's Hospital, The Eighth Affiliated Hospital of Guangxi Medical University, Guigang, China; ^2^Guangxi Botanical Garden of Medicinal Plants, Nanning, China; ^3^Department of Pharmacy, The First Affiliated Hospital of Hainan Medical University, Haikou, China

**Keywords:** COVID-19, diabetes mellitus, bioinformatics, network pharmacology, molecular docking, 1,25-dihydroxyvitamin D

## Abstract

Coronavirus disease 2019 (COVID-19) and diabetes mellitus (DM) are two major diseases threatening human health. The susceptibility of DM patients to COVID-19 and their worse outcomes have forced us to explore efficient routes to combat COVID-19/DM. As the most active form of Vitamin D, 1,25-dihydroxyvitamin D (1,25(OH)_2_D) has been shown a beneficial effect in the treatment of COVID-19/DM. However, the anti-COVID-19/DM mechanisms of 1,25(OH)_2_D remain unclear. In this study, an approach combining network pharmacology and molecular docking was performed to reveal the potential hub target genes and underlying mechanisms of 1,25(OH)_2_D in the treatment of COVID-19/DM. The hub targets and interaction pathways related to 1,25(OH)_2_D were identified by integrating the key 1,25(OH)_2_D-target-signaling pathway-COVID-19/DM networks. Fifteen hub targets of 1,25(OH)_2_D against COVID-19DM were determined, including EGFR, PIK3R1, PIK3CA, STAT3, MAPK1, ESR1, HSP90AA1, LCK, MTOR, IGF1, AR, NFKB1, PIK3CB, PTPN1, and MAPK14. An enrichment analysis of the hub targets further revealed that the effect of 1,25(OH)_2_D against COVID-19/DM involved multiple biological processes, cellular components, molecular functions and biological signaling pathways. Molecular docking disclosed that 1,25(OH)_2_D docked nicely with the hub target proteins, including EGFR, PIK3R1, and PIK3CA. These findings suggested that the potential mechanisms of 1,25(OH)_2_D against COVID-19/DM may be related to multiple biological targets and biological signaling pathways.

## Introduction

The coronavirus disease 2019 (COVID-19) is caused by the severe acute respiratory syndrome coronavirus type 2 (SARS-CoV-2), which brings an unprecedented threat to human health all over the world. As of September 24, 2022, the SARS-CoV-2 had infected approximately 612 million people and had caused at least 6,513 thousand deaths around the world, and both counts are increasing daily. Currently, there are few vaccines, including ChAdOx1 nCoV-19, mRNA-1273, BNT162b2 and CoronaVac, that effectively prevent hundreds of thousands of deaths from SARS-CoV-2. Nevertheless, it was reported that the vaccination program was associated with new vaccine-related adverse reactions, such as hypersensitivity myocarditis (1, 2), Bell's palsy (3), thrombosis and immune thrombocytopenia (4). SARS-CoV-2 attacks cells in the upper and lower airways and then promptly replicates in the infected cells, accompanied by the induction of large amounts of proinflammatory cytokines and chemokines, leading to the so-called cytokine tornado (5). Hence, respiratory symptoms, such as alveolar edema, acute respiratory distress syndrome and vascular leakage, may generally occur in COVID-19 patients. Diabetes mellitus (DM) is a widespread metabolic and endocrine disease, which is likewise another major threat to human health (6). In 2021, the number of DM patients aged 20–79 is estimated to be 536.6 million, and more than 6.7 million died from DM-related causes worldwide (7). Epidemiological investigation demonstrates that DM ranks second among the comorbidities of COVID-19 and that patients with DM are markedly predisposed to SARS-CoV-2 infection (8, 9). In addition, DM is also one of the risk factors most associated with worse outcomes in COVID-19 patients (10, 11). Consequently, there is a crying need to explore effective treatments for COVID-19 patients, particularly for DM patients infected with SARS-CoV-2, which may contribute to improving patient prognosis and survival.

**Figure 1:**
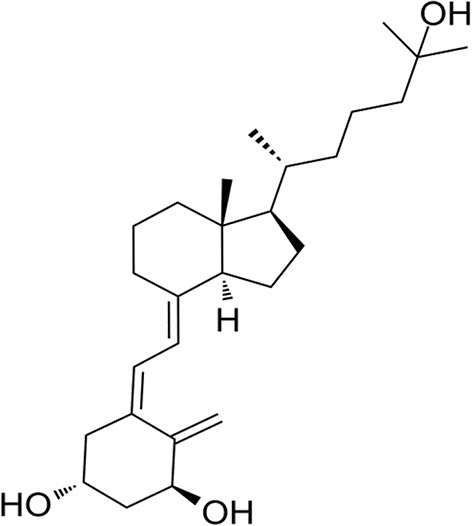
The 2-dimensional structure of 1,25-dihydroxyvitamin D.

Vitamin D (VD), a fat-soluble vitamin, has been found to be associated with many diseases and VD supplementation has become one of the most common nutritional treatments for these diseases around the world. VD is originally world-renowned for regulating calcium and phosphorus metabolism and promoting bone tissue growth. Recent evidence has revealed that the benefits of VD exceed its effects on the skeletal system, reporting on the endocrine and immune systems (5, 12). Currently, studies have shown that VD has a beneficial effect in altering the progression and severity of DM (13, 14) and COVID-19 patients (15–17) and preventing DM patients from being contaminated with SARS-CoV-2 (18, 19). VD deficiency has been implicated in the pathogenesis of obesity and diabetes, affecting insulin secretion (20, 21) and exacerbating inflammatory states (22). Besides, VD may modulate various pathways of the immune system to fight against COVID-19, including inhibiting entry and replication of SARS-CoV-2, increasing the concentrations of anti-inflammatory cytokines, decreasing the level of proinflammatory cytokines, accelerating the production of natural antimicrobial peptides, and activating defense cells that destroy SARS-CoV-2 (23). Furthermore, VD deficiency is also a risk factor contributing to people infected with SARS-CoV-2 (24). VD is inherently inactive and must be further metabolized into an active form for biological effects by the enzymes vitamin D-25-hydroxylase in the liver and 25-hydroxyvitamin D-1-hydroxylase in the kidney (25). Serves as the most active form of VD, 1,25-dihydroxyvitamin D (1,25(OH)_2_D) (Figure 1) exerts its biological activities by binding with the target receptor in the target tissue and then leads to up- or down-regulation of the related genes (26). Studies have shown that patients who take 1,25(OH)_2_D supplementation have an improvement in oxygenation (27), and lower risks of SARS-CoV2 infection, COVID-19 severity and mortality (28). In addition, one study showed that 1,25(OH)_2_D exhibits significant potent activity against SARS-CoV-2 in human nasal epithelial cells and human hepatoma cells *in vitro* (29). It was also reported that lower 1,25 (OH)_2_D levels was significantly associated with DM prevalence in US middle-aged Caucasian men and women (30). Moreover, studies also shown that 1,25(OH)_2_D supplementation decreased the increase in glycemia, and increased insulin secretion (31, 32), and attenuated diabetic cardiomyopathy (33). Furthermore, there are reports showing that 1,25(OH)_2_D exerts its anti-diabetic effect by ameliorating inflammation (34) and exerts its anti-microbial effect in a variety of bacteria, viruses and pathogens by regulating the innate and adaptive immune response (5). However, the role of 1,25(OH)_2_D against COVID-19/DM is still unknown. Therefore, the potential targets and mechanisms of 1,25(OH)_2_D against COVID-19/DM are needed to be further studied.

Network pharmacology, serves as a drug development tool, plays a crucial role in predicting potential targets and revealing underlying mechanisms in clinical disease therapy (35). In this paper, we aimed at exploring the therapeutic action of 1,25(OH)_2_D against COVID-19/DM and revealing its pharmacological pathways by combining network pharmacological analysis with molecular docking technology.

## Materials and methods

### Collection of 1,25(OH)_2_D related genes

**Figure 2:**
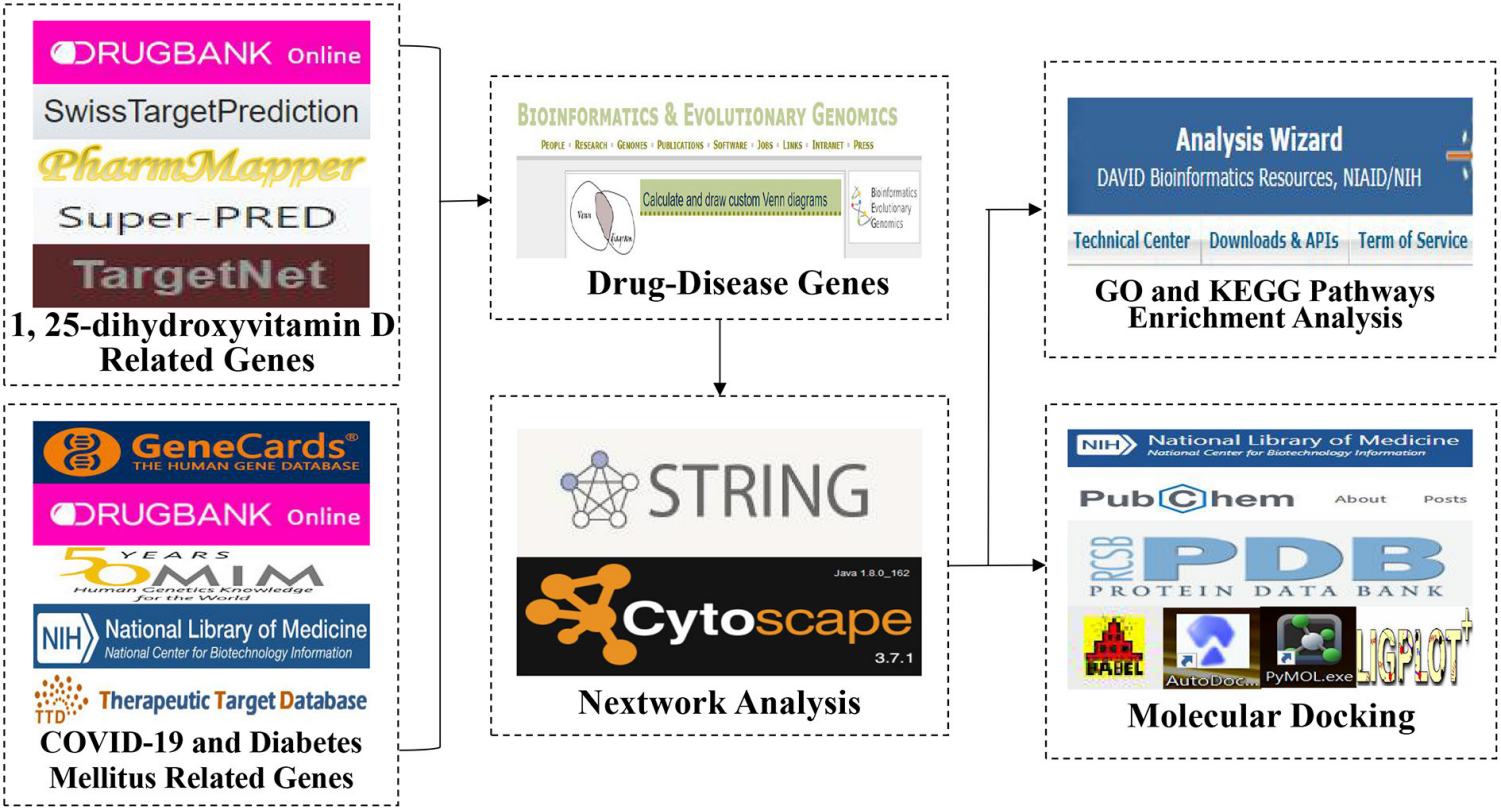
The flow-process diagram of the study.

The flow-process diagram of the study was shown in Figure 2. The genes related to 1,25(OH)_2_D were gained from accessible online public databases including the Drugbank database (36) and the Swiss Target Prediction database (37). In addition, we also obtained the genes related to 1,25(OH)_2_D from the PharmMapper database (38), the SuperPred database (39) and the TargetNet database (40) with a score ≥ 0.6. The protein names were converted to their gene names by utilizing the Uniprot database (41). We accessed the above databases on August 14, 2022.

### Identification of DM/COVID-19 related genes

Further, the genes of DM and COVID-19 were identified by searching on the publicly accessible online databases on August 15, 2022. With the key words including “severe acute respiratory syndrome coronavirus 2,” “SARS-CoV-2,” “coronavirus COVID-19,” “coronavirus Disease 2019,” “COVID-19,” “diabetes mellitus” and “diabetes,” the genes related to COVID-19 and DM were gained from the GeneCards database (https://www.genecards.org/) (42) with a score ≥ 10. Moreover, we also searched the Drugbank database (36), the Online Mendelian Inheritance in Man (OMIM) database (43), the National Center for Biotechnology Information (NCBI) database and the Therapeutic Target database (TTD) (44) for DM and COVID-19 related genes. All the genes related to 1,25(OH)_2_D, COVID-19 and DM were obtained and imported into a free online website (http://bioinformatics.psb.ugent.be/webtools/Venn/) to predict the potential targets of 1,25(OH)_2_D against COVID-19/DM were confirmed. Eventually, a group of mutual genes was acquired.

### Construction of interaction network

The mutual genes were uploaded to the STRING database to construct protein–protein interaction (PPI) network, and then a tab separated values (TSV) file was obtained with a confidence value of interaction score ≥ 0.9. Further, the TSV file was imported into the Cytoscape software (Version 3.7.1) to visualize the PPI network of the identified genes. Furthermore, the Cytoscape software was also executed to select the hub targets of the identified genes by utilizing the “cytoHubba” function.

### Gene ontology and Kyoto Encyclopedia of genes and genomes pathways enrichment analysis

The hub targets were imported into the DAVID database (45) for enrichment analysis involving Gene Ontology and Kyoto Encyclopedia of Genes and Genomes pathways enrichment analysis. Ultimately, the GO and KEGG enrichment data related to hub targets were imported into an online tool for visualized analysis including histogram, bubble chart and circle diagram.

### Construction of multiple network relationships

The hub targets data in the GO and KEGG pathways of 1,25(OH)_2_D against COVID-19/DM were uploaded to the Cytoscape software to establish a multiple interaction network diagram of drug–target–GO and KEGG pathway–disease.

### Molecular docking

Furthermore, the binding affinities of the hub target proteins to 1,25(OH)_2_D were confirmed by using molecular docking imitation *in silico*. The 3-dimensional structure file of 1,25(OH)_2_D was gained from the PubChem database (https://pubchem.ncbi.nlm.nih.gov/), which was further converted to mol2 file by applying the Openbabel software (vision 2.4.1). The mol2 file was imported into the AutoDock Vina program to add hydrogen, set torsion tree and then convert to pdbqt file. In addition, we obtained the 3D structures of the hub target protein from the Protein Data Bank (PDB) database (https://www.rcsb.org/). With AutoDock Vina software, each protein was processed with a few steps, including hydrogen addition and water deletion, and then it was saved as pdbqt files. Grid boxes in the AutoDock Vina software were performed to set the active docking center to include all residues surrounding the original ligand. Finally, the setting of the docking parameters was evaluated by calculating the root mean square deviation (RMSD) of the docked ligand molecule from the original ligand molecule by Pymol software. An RMSD≤4 Å was commonly served as the threshold for matching the docking ligand configuration and the original ligand configuration. Moreover, the docking results were imported into the LigPlot^+^ software (version 2.2.5) to analyze the hydrophobic interactions between ligands and target proteins.

## Results

### Identification of 1,25(OH)_2_D and DM/COVID-19 related genes

**Figure 3:**
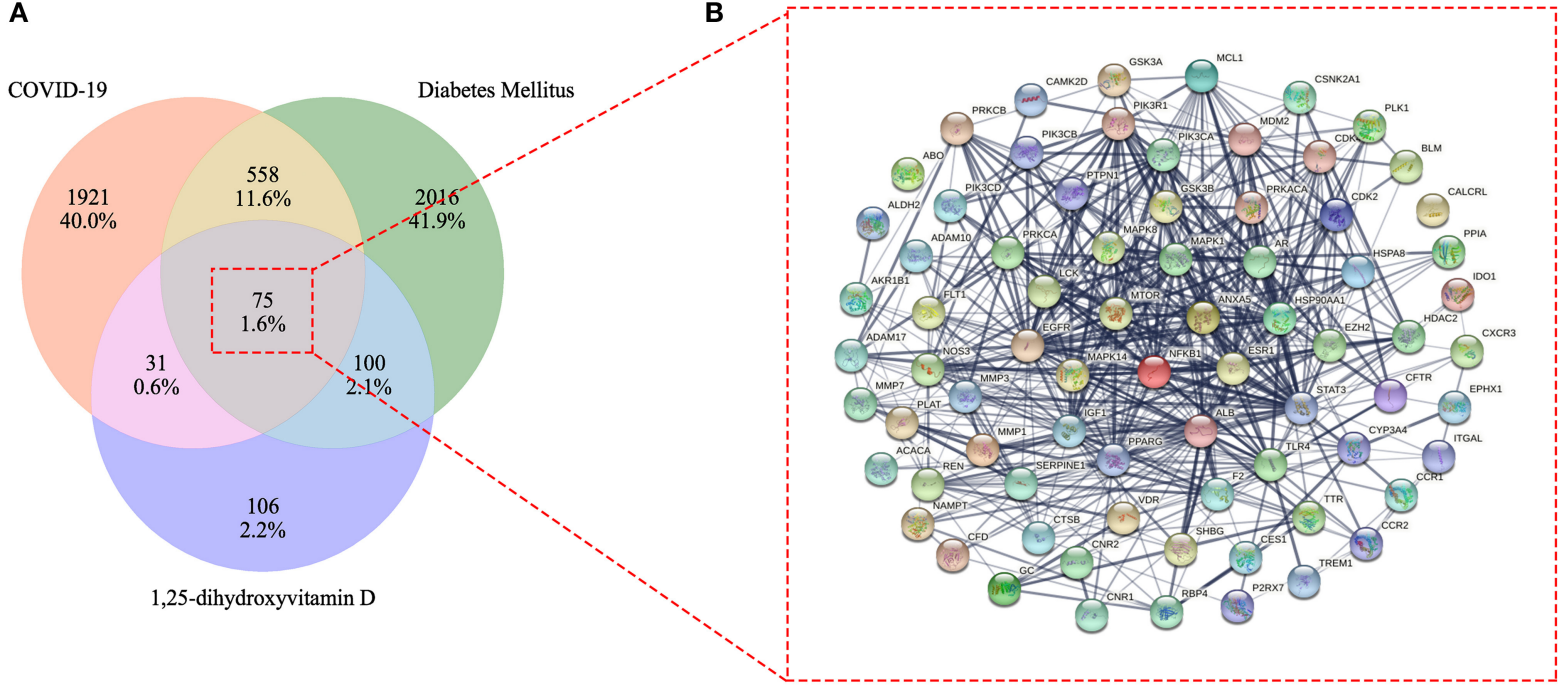
Venn diagram of 1,25-dihydroxyvitamin D effects against COVID-19 and diabetes mellitus (DM) **(A)**. Mutual target genes of 1,25-dihydroxyvitamin D and COVID-19/DM for constructing a protein–protein interaction network **(B)**.

After excluding duplicates, a total of 312 1,25(OH)_2_D related genes were obtained. Furthermore, a total of 2,585 COVID-19 related target genes and 2,749 DM related target genes were identified after eliminating duplicates in each cluster (Supplementary Table S1). Eventually, we identified 75 overlapped genes in these three clusters, which were gained by processing with the Venn diagram. Simultaneously, the overlapped genes were further used to construct their interactional network (Figure 3).

### PPI network and hub targets

**Figure 4:**
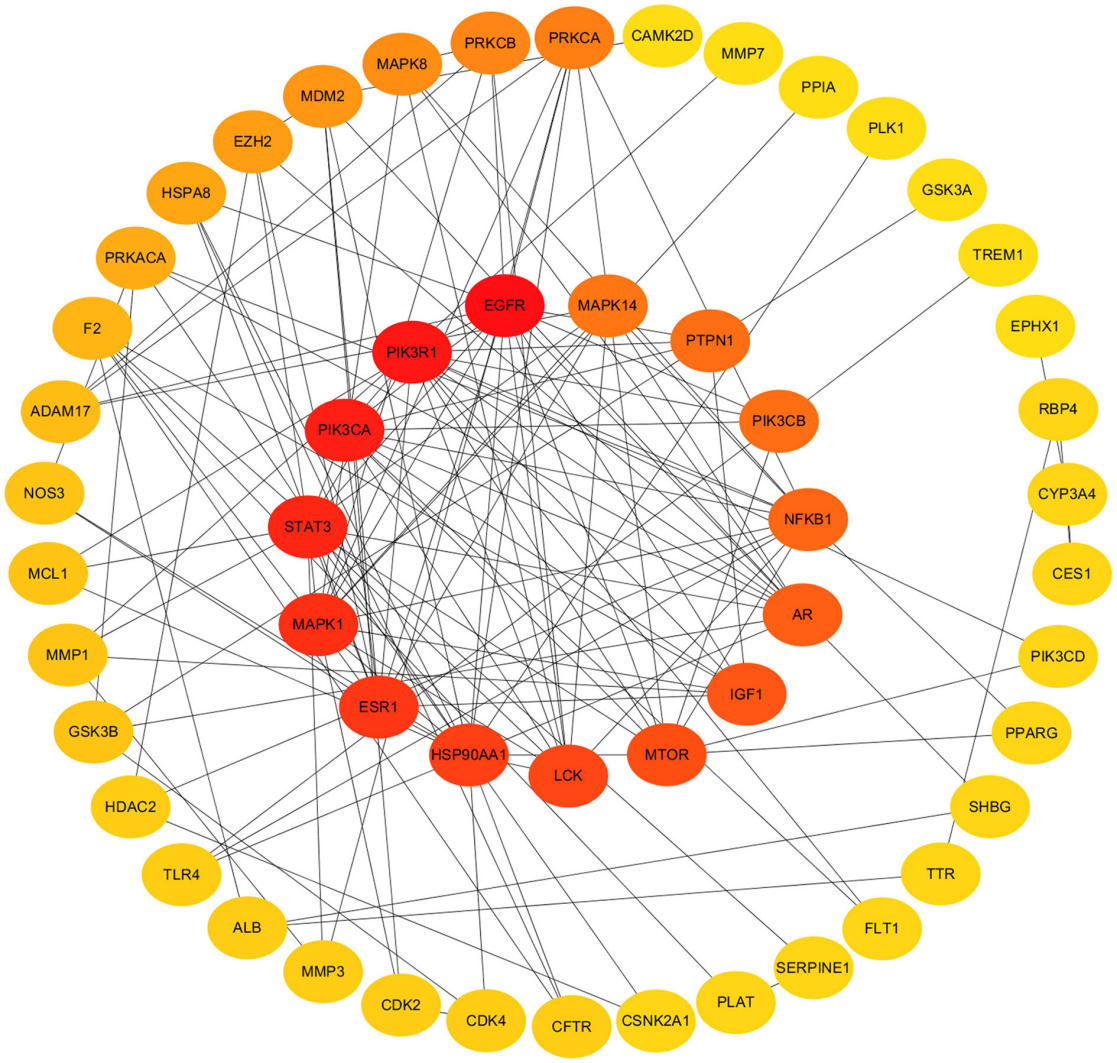
Protein–protein interaction networks and hub target genes. Hub target genes revealed in inner track. The darker the color, the higher the score.

In order to construct PPI network and determine the topological parameters of 1,25(OH)_2_D against COVID-19/DM, the mapped cross-proteins were inputted to the Cytoscape software. Further, the score of maximal clique centrality (MCC) method was applied to determine the hub targets. Finally, the top fifteen targets with a score > 20 were considered as the hub targets, including EGFR, PIK3R1, PIK3CA, STAT3, MAPK1, ESR1, HSP90AA1, LCK, MTOR, IGF1, AR, NFKB1, PIK3CB, PTPN1, and MAPK14 (Figure 4; Supplementary Table S2).

### GO and KEGG pathway enrichment analysis

**Figure 5:**
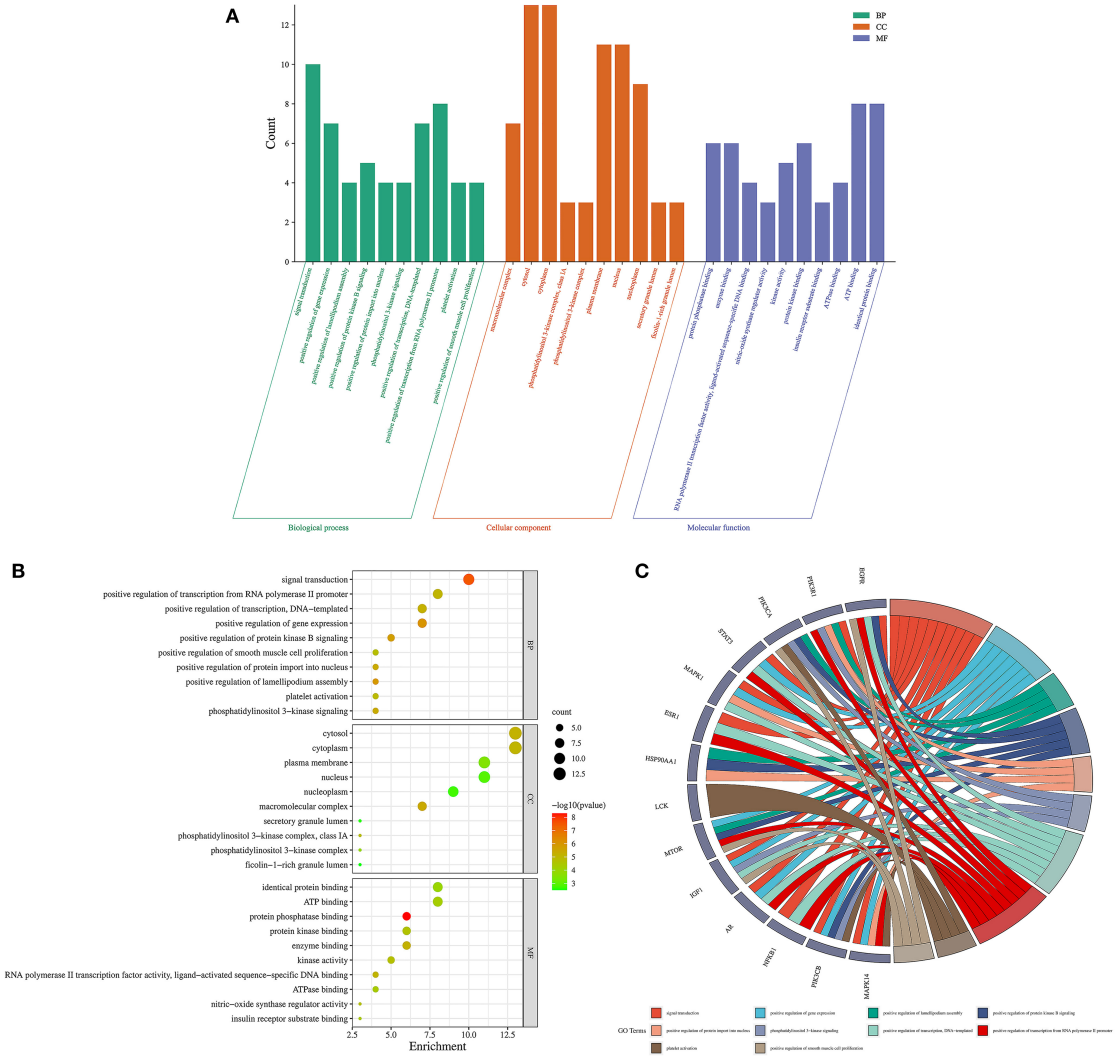
Gene ontology enrichment analysis findings in 1,25-dihydroxyvitamin D against COVID-19/DM. Bar charts of biological processes, cellular components, and molecular functions **(A)**, bubble chart of biological processes, cellular components, and molecular functions **(B)**, circle chart of hub targets and biological processes **(C)**.

**Figure 6:**
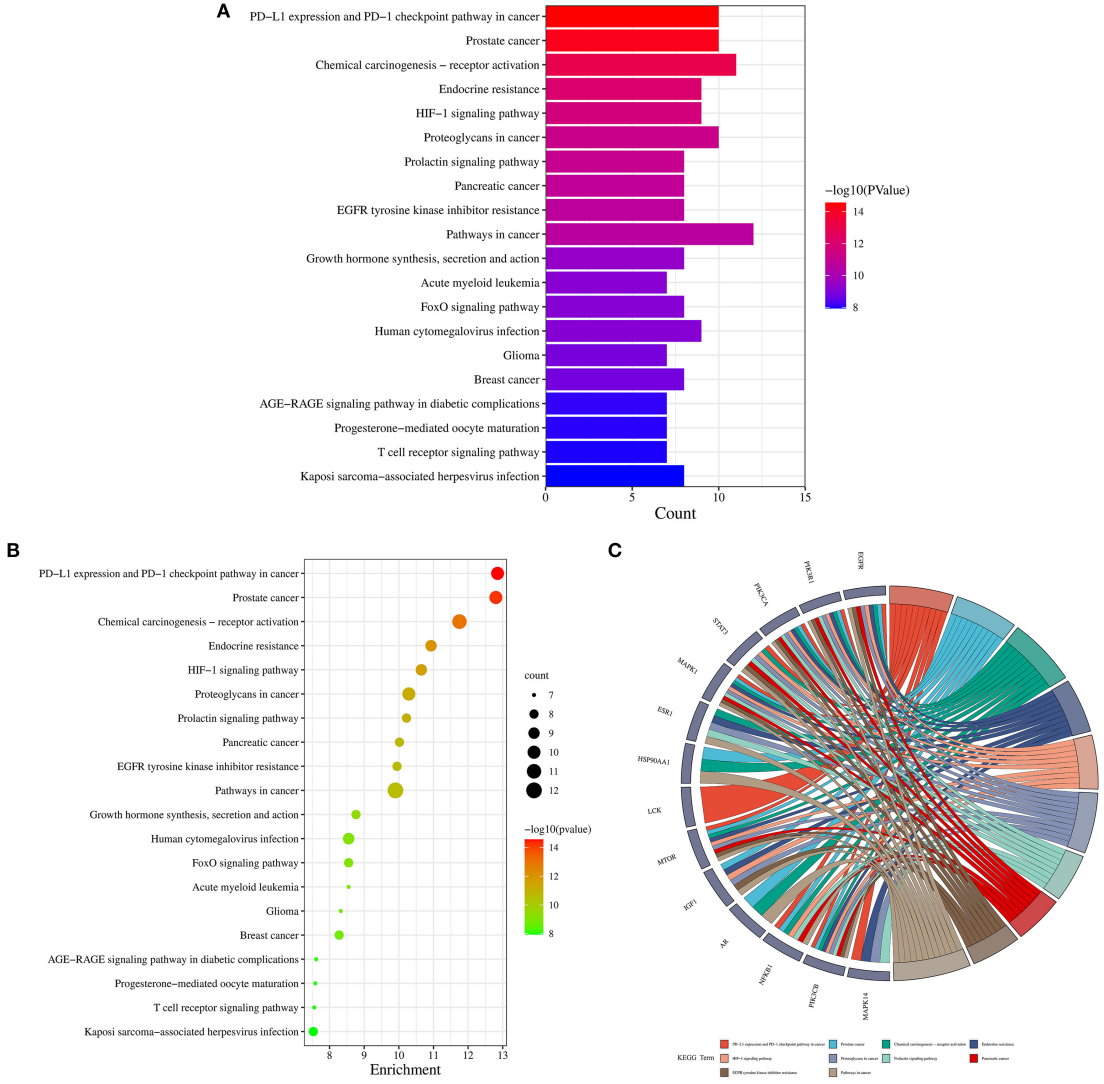
Kyoto Encyclopedia of genes and genomes enrichment analysis findings in 1,25-dihydroxyvitamin D against COVID-19/DM. Bar chart of KEGG pathways **(A)**, bubble chart of KEGG pathways **(B)**, circle chart of hub targets and KEGG pathways **(C)**.

Firstly, the enrichment analysis revealed that the biological processes (BPs) of hub targets were predominantly implicated in signal transduction, positive regulation of gene expression, positive regulation of lamellipodium assembly, positive regulation of protein kinase B signaling, positive regulation of protein import into nucleus, phosphatidylinositol 3-kinase signaling, positive regulation of transcription, DNA-templated, positive regulation of transcription from RNA polymerase II promoter, platelet activation, positive regulation of smooth muscle cell proliferation, transcription from RNA polymerase II promoter, positive regulation of cell migration, negative regulation of gene expression, negative regulation of gene expression, response to muscle stretch, phosphorylation, regulation of multicellular organism growth, positive regulation of glucose import, negative regulation of apoptotic process, and phosphatidylinositol-mediated signaling. In addition, the hub targets related to cellular components (CCs) were principally involved in macromolecular complex, cytosol, cytosol, phosphatidylinositol 3-kinase complex, class IA, phosphatidylinositol 3-kinase complex, plasma membrane, nucleus, nucleoplasm, secretory granule lumen, ficolin-1-rich granule lumen, membrane, perinuclear region of cytoplasm, glutamatergic synapse, chromatin, extracellular region, and mitochondrion. Moreover, the hub targets associated with molecular functions (MFs) were primarily engaged in protein phosphatase binding, enzyme binding, RNA polymerase II transcription factor activity, ligand-activated sequence-specific DNA binding, nitric-oxide synthase regulator activity, kinase activity, protein kinase binding, insulin receptor substrate binding, ATPase binding, ATP binding, identical protein binding, insulin receptor binding, transcription factor binding, transcription coactivator binding, phosphotyrosine binding, DNA binding, protein serine/threonine kinase activity, protein binding, phosphatidylinositol 3-kinase activity, chromatin binding, and transcriptional activator activity, RNA polymerase II transcription regulatory region sequence-specific binding (Figures 5A–C; Supplementary Tables S3–5). Finally, a total of 120 KEGG pathways related to hub targets were identified as well (*p* < 0.05), including PD-L1 expression and PD-1 checkpoint pathway in cancer, prostate cancer, chemical carcinogenesis-receptor activation, endocrine resistance, HIF-1 signaling pathway, proteoglycans in cancer, prolactin signaling pathway, pancreatic cancer, EGFR tyrosine kinase inhibitor resistance, pathways in cancer, growth hormone synthesis, secretion and action, acute myeloid leukemia, FoxO signaling pathway, human cytomegalovirus infection, glioma, breast cancer, AGE-RAGE signaling pathway in diabetic complications, progesterone-mediated oocyte maturation, T cell receptor signaling pathway, and Kaposi sarcoma-associated herpesvirus infection (Figures 6A–C; Supplementary Table S6).

### Visualization of multiple network relationships

**Figure 7:**
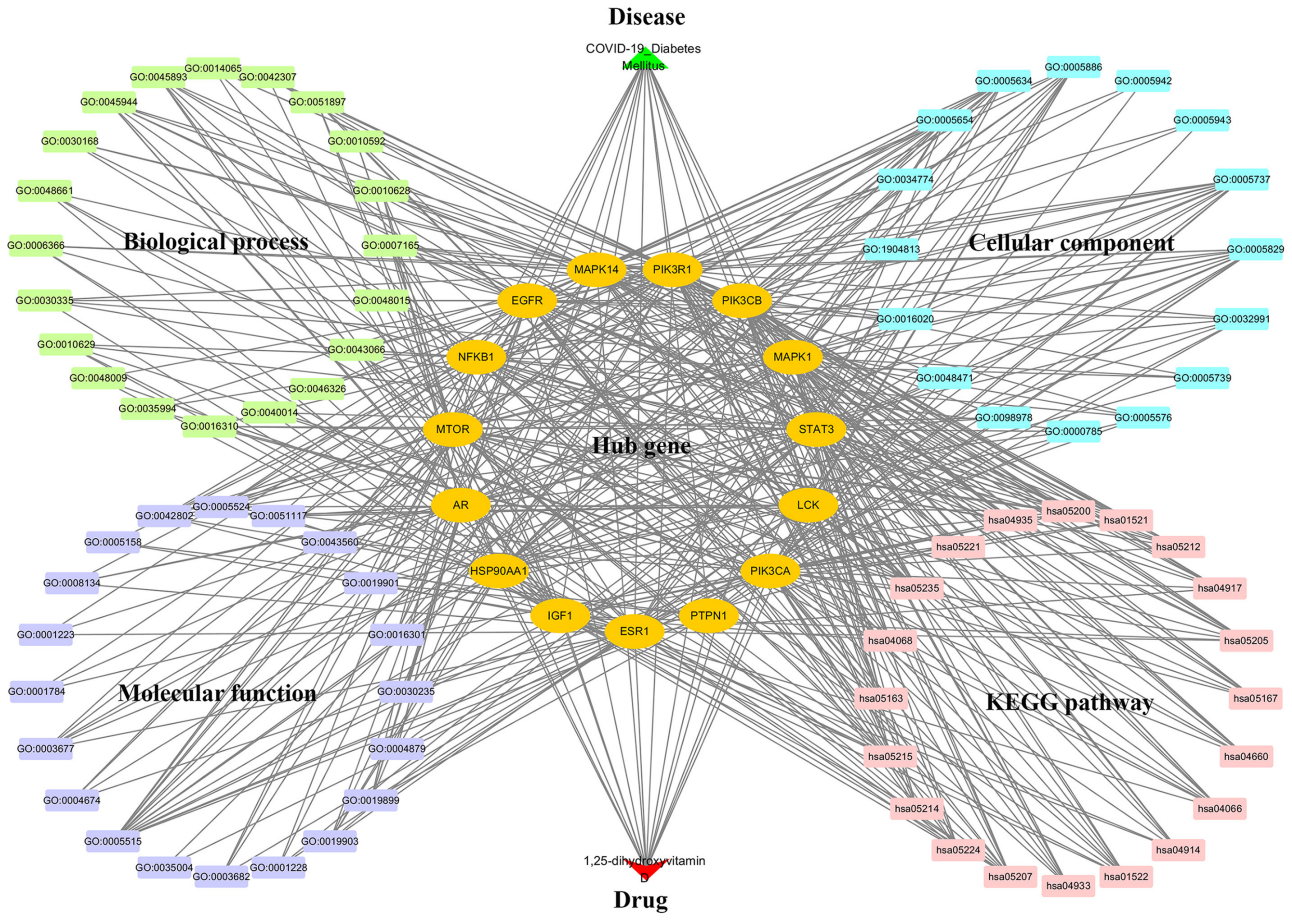
1,25-dihydroxyvitamin D–targets–GO and KEGG pathways–COVID-19/DM network diagram.

A multiple interaction network diagram of drug–target–GO and KEGG pathway–disease was constructed by using the Cytoscape software, revealing that the role of 1,25(OH)_2_D against COVID-19/DM was involved in multiple BPs, CCs, MFs, and KEGG pathways (Figure 7).

### Molecular docking analysis

**Figure 8:**
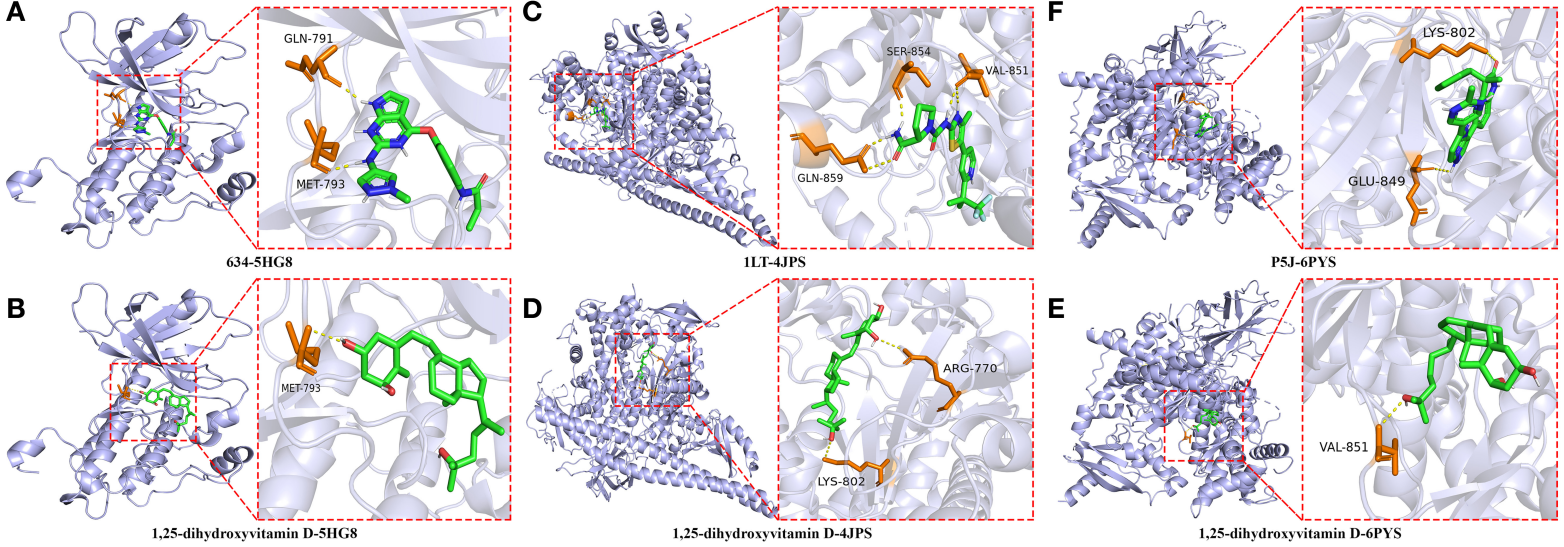
Molecular docking details of the original ligands and 1,25-dihydroxyvitamin D to the target protein. 634-5HG8 **(A)**, 1,25-dihydroxyvitamin D-5HG8 **(B)**, 1LT-4JPS **(C)**, 1,25-dihydroxyvitamin D-4JPS **(D)**, P5J-6PYS **(E)**, 1,25-dihydroxyvitamin D-6PYS **(F)**.

The highest scoring hub targets, including EGFR (PBD ID: 5HG8), PIK3R1 (PBD ID: 4JPS) and PIK3CA (PBD ID: 6PYS), were selected for docking validation. The parameters in the grid box of EGFR were set as 12.724, −3.444, and −30.049 for the center-x-y-z and 15, 15, and 15 for the size-x-y-z. The original ligand 634 docked with the 5HG8 protein by combining with the amino acid residues GLN-791 (2.0 Å) and MET-793 (2.0 Å) (Figure 8A). The RMSD and free bonding energy of the 634 were 0.221 Å and −8.5 kcal/mol, respectively. The hydrogen bond formation between 1,25(OH)_2_D and the 5HG8 protein involved the amino acid residue MET-793 (3.0 Å) (Figure 8B) with −7.1 kcal/mol free bonding energy. In addition, the parameters in the grid box of PIK3R1 were set as −1.166, −8.926, and 16.981 for center-x-y-z and 15, 15, and 17.25 for size-x-y-z. The original ligand 1LT docked with the 4JPS protein by bonding with the amino acid residues SER-854 (1.9 Å), VAL-851 (2.1 Å, 2.5 Å) and GLN-859 (1.9 Å, 2.9 Å) (Figure 8C). The RMSD and free bonding energy of the 1LT were 0.095 Å and −10.3 kcal/mol, respectively. The hydrogen bond formation between 1,25(OH)_2_D and the 4JPS protein included the amino acid residues ARG-770 (3.2 Å) and LYS-802 (3.0 Å) (Figure 8D) with −7.7 kcal/mol free bonding energy. Furthermore, the parameters in the grid box of PIK3CA are set as −19.077, 11.460, and 28.185 for center-x-y-z and 15, 15, and 15 for size-x-y-z. The original ligand P5J docked with the 6YPS protein by docking with the amino acid residues LYS-802 (2.9 Å) and GLU-849 (2.9 Å) (Figure 8E). The RMSD and free bonding energy of the P5J were 0.006 Å and −11.3 kcal/mol, respectively. The hydrogen bond formation between 1,25(OH)_2_D and the 6YPS protein involved the amino acid residue VAL-851 (3.1 Å) (Figure 8F) with −8.1 kcal/mol free bonding energy.

**Figure 9:**
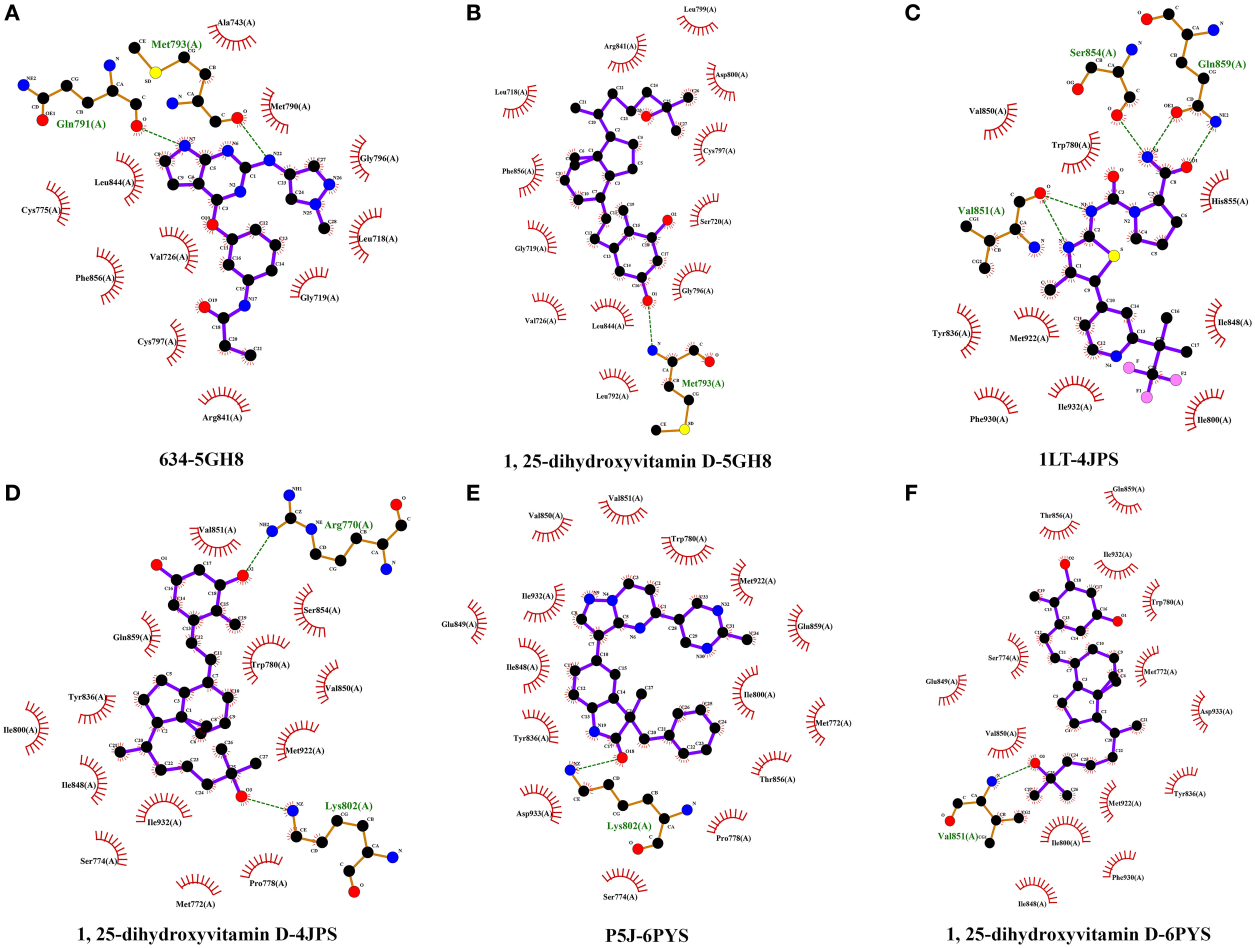
The hydrophobic interactions of original ligands and 1,25-dihydroxyvitamin D with the target protein. Hydrophobic interactions are indicated by red opposite arcs. Hydrogen bonds are shown as green dashed lines. 634-5HG8 **(A)**, 1,25-dihydroxyvitamin D-5HG8 **(B)**, 1LT-4JPS **(C)**, 1,25-dihydroxyvitamin D-4JPS **(D)**, P5J-6PYS **(E)**, 1,25-dihydroxyvitamin D-6PYS **(F)**.

Furthermore, we performed an analysis of the hydrophobic interactions between the ligands and the target proteins by using the LigPlot^+^ software. The hydrophobic amino acids of the docking domain of the 5GH8 protein, which engage in the hydrophobic interactions with the ligand 634, are Cys775, Leu844, Phe856, Val726, Cys797, Arg841, Ala743, Met 790, Gly796, Leu718, and Gly 719 (Figure 9A), and with 1,25(OH)_2_D are Arg841, Leu718, Phe856, Gly719, Val726, Leu844, Leu792, Leu799, Asp800, Cys797, Ser720, and Gly796 (Figure 9B). Similarly, the ligand 1LT was found to interact with the docking domain of the 4JPS protein through nine hydrophobic amino acids, including Val850, Trp780, Try836, Met 922, Phe930, Ile932, His855, Ile848, and Ile800 (Figure 9C), and 1,25(OH)_2_D was found to interact more effectively with the docking domain of the 4JPS protein through thirteen hydrophobic amino acids, including Val851, Gln859, Ile800, Tyr836, Ile848, Ser774, Ile932, Met772, Pro778, Ser854, Trp780, Val850, and Met922 (Figure 9D). Equivalently, the hydrophobic interactions between the ligand P5J and the docking domain of the 6PYS protein are through fifteen hydrophobic amino acids, including Val851, Val850, Glu849, Ile932, Ile848, Tyr836, Asp933, Ser774, Trp780, Met922, Gln859, Ile800, Met772, Thr856, and Pro778 (Figure 9E). The hydrophobic interactions between 1,25(OH)_2_D and the docking domain of the 6PYS protein are through fourteen hydrophobic amino acids, including Thr856, Glu849, Ser774, Val850, Gln859, Ile932, Trp780, Met772, Asp933, Tyr836, Met922, Ile800, Phe930, and Ile848 (Figure 9F). From the above results, it is clear that the original ligands and the docked ligands share many hydrophobic amino acids with the target proteins.

## Discussion

Since the first outbreak of COVID-19 in China, a lot of attention has been focused on people with DM due to the worse outcomes among those infected (46). The large amount of proinflammatory cytokines and chemokines formed after SARS-CoV-2 infection may contribute to increasing the risk of poor prognosis in DM patients (5, 46). In this paper, the combination of bioinformatics analysis, network pharmacology and molecular docking was performed to determine the potential targets, biological function and signaling pathways of 1,25(OH)_2_D in the treatment of COVID-19/DM. Firstly, fifteen hub targets of 1,25(OH)_2_D against COVID-19/DM included EGFR, PIK3R1, PIK3CA, STAT3, MAPK1, ESR1, HSP90AA1, LCK, MTOR, IGF1, AR, NFKB1, PIK3CB, PTPN1, and MAPK14 were identified by applying network pharmacology that is a frequently-used approach to pharmaceutical research for multiple diseases included COVID-19 and DM. Previous studies indicated that most of these hub targets may be vital to clarify the therapeutic mechanism of COVID-19 and DM diseases.

EGFR, serves as a member of the epidermal growth factor receptor (HER) family, has physiological functions in regulating epithelial tissue development and homeostasis (47). Numerous cells in the lung infected with SARS-CoV-2 cause EGFR overexpression, which contributes to worsening the pulmonary disease and triggering fibrosis (48). In addition, deletion of EGFR in podocytes also leads to delaying the development of diabetic nephropathy (49). EGFR activation also leads to STAT3 phosphorylation, which can induce inflammatory responses and imbalanced anti-virus adaptive immune responses, inhibit anti-virus interferon responses, and promote M2 macrophage polarization, pulmonary fibrosis, and thrombosis (50). Besides, activation of STAT3 not only contributes to involvement in the development of diabetic insulin resistance in DM (51) but also participates in the progress of diabetes-associated cardiovascular disease, such as myocardial interstitial fibrosis and myocardial infarction (52, 53). The Class I phosphoinositide 3-kinases (PI3Ks) included PIK3R1, PIK3CA, and PIK3CB, are a group of heterodimeric lipid kinases that regulate vital cellular processes involving survival, growth, proliferation, and metabolism (54). Previous studies show that PI3Ks play a crucial role in regulating the development of DM (55–57).

Our results also revealed that the mitogen-activated protein kinase family members, such as MAPK1 and MAPK14, were potential targets of 1,25(OH)_2_D against COVID-19/DM. These two targets can be activated by provoking phosphorylation during changes in the internal environment, including oxidative stress, osmotic viral infection, inflammatory factors and pressure changes (58). ESR1 is a crucial sex factor that provides a protective umbrella to COVID-19 patients by suppressing the immune and inflammatory responses induced by SARS-CoV-2 infection (59) and protects DM patients by improving glycemic homeostasis (60). In addition, recent studies reveal that inhibition of HSP90AA1 activity contributes to reducing SARS-CoV- 2 viral replication and tumor necrosis factor (TNF) mRNA levels (61), and enhancing glucose-stimulated insulin secretion and expressions of genes tightly associated with β-cell function (62). LCK, serves as a cytoplasmic tyrosine kinase, is expressed in natural killer cells and T cells and plays an irreplaceable role in activating T cells. Hence, selective inhibition of LCK expression can lead to immunosuppression (63). MTOR is a key protein in the mTOR pathway that appears to play a crucial role in regulating the immune dysregulation processes underlying COVID-19 in diabetic patients (64). In addition, continuous hyperactivation of MTOR in diabetic myocardium has been demonstrated, which makes MTOR become a potential target in the prevention of diabetes-related cardiovascular disease (53). Current evidence also shows that MTOR activation plays a crucial role in the pathogenesis of insulin resistance in type 2 diabetes mellitus (T2DM) (65, 66). Furthermore, recent studies show that higher IGF1 level is associated with lower incidence of T2DM and lower risk of COVID-19 mortality (64, 67).

In the next section of this paper, the enrichment analysis of hub targets revealed their importance in diverse biological processes. In this paper, we focused on the functions and signaling pathways involved in the pathogenesis of COVID-19/DM. The signaling pathways, including HIF-1 signaling pathway, FoxO signaling pathway, T cell receptor signaling pathway, PI3K-Akt signaling pathway and so on (Figure 4; Supplementary Table S6), were found to be targeted by 1,25(OH)_2_D. HIF-1 acts as a regulator of oxygen homeostasis and has been reported to enhance immunity by regulating the tasks of neutrophils, lymphocytes, dendritic cells and macrophages, which are disrupted in COVID-19 infections, contributing to hypoxic conditions (68). Research has also demonstrated that inhibition or activation of the HIF-1 signaling pathway was associated with β-cell dysfunction, insulin resistance and glucose intolerance (69). The FoxO signaling pathway plays a critical role in multifarious physiological and cellular processes, including cell proliferation, regulation of programmed cell death, cell cycle regulation, and regulation of glycogenolysis and gluconeogenesis. Fang et al. found that FoxO signaling pathway was related to diabetic complications and COVID-19 by analyzing the differentially expressed genes in those patients (70). In addition, the complex molecular mechanism mediated by T cell receptor signaling pathway can cause the activation of T cells, thereby affecting the immune response of the body (71). It was reported that T cell receptor signaling pathway might represent the main pathological mechanism of gestational diabetes mellitus (72). Furthermore, the PI3K-Akt signaling pathway has been confirmed to be participated in a variety of cellular processes including synthesis, glucose transport and breakdown in DM (73), and also to be involved in the pathogenesis of pulmonary fibrosis and immune response process of the host cell to resist viral invasion (74).

Finally, the interaction of 1,25(OH)_2_D and its targets was identified by conducting molecular docking. Molecular docking is a frequently used tool for predicting possible interactions between two molecules and is a potent approach for structure-based drug discovery (75). In this paper, we applied molecular docking to analyze the binding affinity of 1,25(OH)_2_D to EGFR, PIK3R1, and PIK3CA. Our results revealed that 1,25(OH)_2_D has a strong binding affinity with these three targets by forming of hydrogen bonds and hydrophobic interactions, indicating the drug-protein interaction and the anti-COVID-19/DM activity of 1,25(OH)_2_D. Nonetheless, all of the findings in this paper were predicted only by integrated bioinformatics analysis and *in silico* approaches. However, the current network information technology is not comprehensive enough, and the real-time update and accuracy of these databases need to be improved. Thus, all of the findings obtained in this paper should be further validated from a pharmacodynamic perspective, and preclinical studies are needed to account for the multiple biological targets and biological signaling pathways of 1,25(OH)_2_D in the treatment of COVID-19/DM.

## Conclusion

In summary, our results identified the possible hub targets and underlying pharmacological mechanisms of 1,25(OH)_2_D in the treatment of COVID-19/DM by applying network pharmacology and molecular docking. The hub targets including EGFR, PIK3R1, PIK3CA, STAT3, MAPK1, etc., and the biological signaling pathways including HIF-1 signaling pathway, FoxO signaling pathway, T cell receptor signaling pathway, PI3K-Akt signaling pathway, etc. involved in these targets are considered to play a critical role in 1,25(OH)_2_D against COVID-19/DM. Therefore, the effect of 1,25(OH)_2_D in the treatment of COVID-19/DM may be achieved by targeting multiple biological targets and biological signaling pathways. However, preclinical validation is required to confirm these findings in further studies.

## Data availability statement

The original contributions presented in the study are included in the article/Supplementary material, further inquiries can be directed to the corresponding authors.

## Author contributions

FZ, ZY, and CW conceived and designed the study and drafted the manuscript. FZ, YX, and CT performed the data analysis and drew charts and graphs. FZ and CT performed the bioinformatics and statistical analyses. All authors contributed to this paper and approved the published version of the manuscript.

## Funding

This study was funded by the National Natural Science Foundation of China (Grant No. 81960705).

## Conflict of interest

The authors declare that the research was conducted in the absence of any commercial or financial relationships that could be construed as a potential conflict of interest.

## Publisher's note

All claims expressed in this article are solely those of the authors and do not necessarily represent those of their affiliated organizations, or those of the publisher, the editors and the reviewers. Any product that may be evaluated in this article, or claim that may be made by its manufacturer, is not guaranteed or endorsed by the publisher.
